# Effects of Negative Emotions and Cognitive Characteristics on Impulse Buying During COVID-19

**DOI:** 10.3389/fpsyg.2022.848256

**Published:** 2022-04-25

**Authors:** Yongjuan Yu

**Affiliations:** School of Finance and Economics, Yangtze Normal University, Chongqing, China

**Keywords:** anxiety, depression, intolerance of uncertainty, cognitive flexibility, impulse buying

## Abstract

The COVID-19 pandemic has seriously disrupted the individual buying habits along with their consumption patterns. Previous studies indicated that anxiety and depression were related to impulse buying. However, no research has explored the mechanism possibly underlying the association between anxiety, depression, and impulse buying. Based on the regulatory focus theory and the emotion-cognition-behavior loop, this study aimed to examine the impacts of negative emotions on impulse buying and the mediating role of cognitive characteristics during the COVID-19 pandemic. In April 2021, 734 Chinese undergraduates were recruited by cluster sampling and they completed self-report measures of anxiety, depression, intolerance of uncertainty, cognitive flexibility, and impulse buying. Results showed that impulse buying was positively associated with anxiety, depression, and intolerance of uncertainty, while it was negatively associated with cognitive flexibility. Cognitive flexibility fully mediated the effects of anxiety and depression on cognitive facet of impulse buying. Meanwhile, intolerance of uncertainty fully mediated the effects of anxiety and depression on affective facet of impulse buying. Overall, this study shows that different pathways can explain how anxiety and depression exacerbate two aspects of impulse buying, and it highlights the importance of cognitive characteristics for the link between negative emotions and impulse buying. Intervention programs should focus on increasing cognitive flexibility and tolerance to uncertainty of high-risk individuals, so as to strengthen their adaptive purchase behaviors.

## Introduction

The 2019 novel coronavirus disease (COVID-19) inevitably has a sustained and accumulating impact on individuals’ physical and mental health (Balsamo and Carlucci, 2020; Liang et al., 2020; Çelik and Köse, 2021; Yu et al., 2021), as well as social and economic activities throughout the world (Carlucci et al., 2020; Clemens et al., 2020), which also enables real-life research on threat-and-defense processes. It should be noted that the COVID-19 pandemic has brought many uncertainties and challenges to people (Denfeld et al., 2020). In terms of consumption, the implementation of social isolation and blockade has completely disrupted consumers’ buying habits and consumption patterns (Sheth, 2020; Naeem, 2021). Individuals have been involved in impulse buying in response to the prolonged isolation and uncertainty (Ahmed et al., 2020; Çelik and Köse, 2021).

After a major disaster, the consequences of changes in individual consumption behavior are often referred to as “impulse buying,” “compulsive buying,” or “hoarding” (Çelik and Köse, 2021; Naeem, 2021). In this study, the word “impulse buying” was used. Impulse buying is defined as a spontaneous, unreflective, and unplanned purchase (Lin et al., 2018). Impulse buying was a common occurrence across the world during the COVID-19 pandemic and led to irrational hoarding and waste of social resources, thereby resulting into disorder or even paralysis of the retail industry (Gupta et al., 2021; Wang et al., 2021). According to Verplanken and Herabadi (2001), impulse buying includes two core dimensions, namely cognitive and affective facets. The cognitive facet refers to the lack of careful planning and consideration for the purchase of the impulsively bought product. While the affective facet, including feelings of pleasure, excitement, compulsion, lack of control, and regret, may occur prior to, simultaneously with, or after an unplanned purchase. Verplanken and Herabadi (2001) pointed out that the two components of impulse buying tendency have different bases in individuals’ personalities. Therefore, the current study examined the cognitive and affective facets of impulse buying tendency, respectively.

The spontaneous and powerful urge to buy is found to be related to many factors, which can be divided into two categories: external situational factors and internal personal factors (Haq and Abbasi, 2016). Previous studies conducted in the Chinese context (Akram et al., 2018a,b) have examined the impacts of external factors, such as website quality, credit card use, and sales promotion, on impulse buying behaviors. A recent study conducted in a multi-country setting examined effects of external stimuli such as limited quantity scarcity and limited time scarcity on panic buying during COVID-19 (Islam et al., 2021). However, it still remains unknown how internal factors affect individual pattern of impulse buying. In order to avoid consumers’ panic and irrational consumption during COVID-19, this study focused on internal influencing factors of impulse buying behavior from an individual perspective.

## Theoretical Framework and Hypotheses Development

Empirical studies have pointed out that impulsive purchase can be driven by emotional stimuli (Leverin and Liljander, 2006; Ahmed et al., 2020), and it is closely related to negative emotions (Silvera et al., 2008). It has been found that individuals who had more anxiety or depression symptoms were more likely to exhibit higher levels of impulsivity (Yu et al., 2020). A diary study of impulsive buying during COVID-19 demonstrated that daily anxiety contributed to daily impulsive buying (Xiao et al., 2020). Silvera et al. (2008) also reported that high frequency impulse buying function as a form of escape from depression. Accordingly, it is expected that individuals with more negative emotions have a stronger tendency toward impulse buying behaviors. Meanwhile, identifying the mediator of this predictive relationship is of great utility. The cognitive behavior theory proposed by Clark and Fairburn (1997) provides a special perspective to study how anxiety and depression link to impulse buying. Previous studies have found that individual behavior is the result of the interaction of emotion and cognition. In the process of interaction with the outside world, individual repeated experience makes emotion, cognition and related behavior often work through the emotion-cognition-behavior model (Guastella and Dadds, 2009). According to the broaden-and-build theory of positive emotions (Fredrickson, 2001), positive emotions usually promote cognition, activate behavior, and help individuals achieve specific goals. On the contrary, negative emotions (such as depression and anxiety) can easily entrap individuals into a “negative loop,” limit their perception and thinking, and interfere with information interpretation, utilization, and response (Deveney and Deldin, 2006; Yu et al., 2020). Therefore, roles of individual cognitive characteristics in the relationship between negative emotions and impulse buying should be concerned.

As a cognitive predisposition, intolerance of uncertainty can affect the individual’s perception, explanation, and reaction to uncertain situations (Dugas et al., 2004). It makes people hard to accept possible negative events, irrespective of the probability of occurrence (Carleton et al., 2007). Intolerance to uncertainty has been found among individuals diagnosed with anxiety disorder (Morriss et al., 2016), post-traumatic stress disorder, and depression disorder (Hollingsworth et al., 2018). It has been demonstrated that anxiety and depression felt in the face of uncertainty may result in maladaptive behaviors such as impulsive decision making (Satici et al., 2020). However, to the best of our knowledge, no study has been conducted to test the mediating role of intolerance of uncertainty in the relationship between anxiety, depression, and impulse buying.

Contrarily, as an essential factor of executive function, cognitive flexibility refers to the ability to freely shift cognitive sets to perceive or respond to internal and external environment in multiple ways (Rende, 2000; Johnco et al., 2014). A clinical study revealed that cognitively inflexible individuals exhibited more severe impulse symptoms (Chamberlain et al., 2006). Cognitive flexibility allows individuals to redefine their current understanding of the COVID-19 pandemic and reconsider behaviors that helps them mitigate their risk in a challenging context (Kalia et al., 2020). A cross-sectional investigation on 477 participants indicated that cognitive flexibility could mitigate individuals’ impulsivity and proposed that cognitive flexibility served as a mediator between negative emotions and impulsivity (Yu et al., 2020). However, no research to date has explored the mediating role of cognitive flexibility in the association between negative emotions and impulse buying behaviors. Therefore, it was examined in the current study. Solutions to this problem can better illustrate how to reduce individuals’ impulsive behaviors.

**FIGURE 1:**
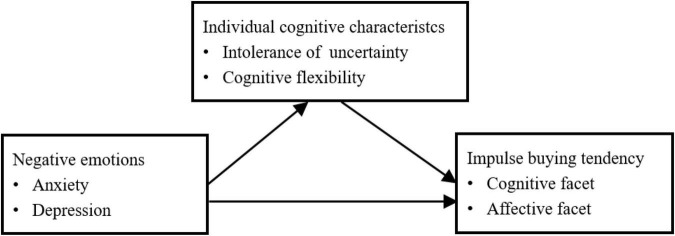
The hypothesized emotion-cognition-behavior model to impulse buying tendency.

According to the cognitive behavior theory (Clark and Fairburn, 1997), an emotion-cognition-behavior framework for impulse buying was developed in the current study (see Figure 1). We assume that negative emotions (anxiety and depression) and cognitive characteristics (intolerance of uncertainty and cognitive flexibility) have significant influences on impulse buying. Meanwhile, negative emotions and cognitive characteristics may interact and exhibit their impacts on impulse buying together. Specifically, anxiety and depression may aggravate individuals’ intolerance of uncertainty and reduce their cognitive flexibility, thereby leading to impulse buying. Accordingly, our hypotheses are as follows:

**Hypothesis 1:** Impulse buying is positively associated with anxiety, depression, and intolerance of uncertainty, while it is negatively associated with cognitive flexibility.

**Hypothesis 2:** Anxiety and depression are positively associated with intolerance of uncertainty and negatively associated with cognitive flexibility.

**Hypothesis 3:** Intolerance of uncertainty and cognitive flexibility mediate the relationships between anxiety, depression, and impulse buying after controlling for related demographic variables.

## Materials and Methods

### Participants and Procedures

This study was approved by the Ethics Committee of Yangtze Normal University. All procedures were carried out in accordance with the Declaration of Helsinki. In April 2021, selected by a cluster sampling technique, 734 undergraduates were recruited from two departments in one university in Chongqing, China. At the time of our investigation, there were still sporadic cases in our country, and the pressure to fight the epidemic has always existed. Prior to the commencement, the purpose and contents of this study were explained to all participants. After signing the written informed consent, all participants completed a set of online questionnaires, including self-reported measures of background information (gender, age, and individual economic status), anxiety, depression, intolerance of uncertainty, cognitive flexibility, and impulse buying.

Of participants, 18 participants with completion time less than 300 s were excluded. The final sample consisted of 716 participants. The ages of these valid participants in our study are from 17 to 25 years (Mean = 20.16, SD = 1.44). There were 133 male students (18.58%) and 583 female students (81.42%). Individual economic status was assessed by monthly disposable living expenses. According to their reports, there were 181 students (25.28%) whose monthly disposable living expenses less than 1000 RMB (bad economic status), 372 students (51.96%) with 1001–2000 RMB monthly disposable living expenses (moderate economic status), and 163 students (22.76%) with more than 2000 RMB monthly disposable living expenses (good economic status).

### Study Measures

#### Anxiety

We adopted the 7-item Generalized Anxiety Disorder Questionnaire (GAD-7; Spitzer et al., 2006; Qu and Sheng, 2015) to measure the anxiety symptoms over the past 2 weeks. For example, “Not being able to stop or control worrying.” Responses ranged from 0 (not at all) to 3 (nearly every day). The GAD-7 has been demonstrated adequate reliability and validity among the Chinese samples (Qu and Sheng, 2015). The Cronbach’s alpha coefficient for GAD-7 was 0.91 in this study.

#### Depression

We used the 9-item Patient Health Questionnaire (PHQ-9; Kroenke et al., 2001; Zhang et al., 2013) to measure depression symptoms in the past 2 weeks. For example, “Little interest or pleasure in doing things.” Participants were asked to rate the items from 0 (not at all) to 3 (nearly every day). Good reliability and validity of PHQ-9 in Chinese adults have been confirmed (Zhang et al., 2013). The Cronbach’s alpha coefficient for PHQ-9 was 0.90 in our sample.

#### Cognitive Flexibility

The 20-item Cognitive Flexibility Inventory (CFI, Dennis and Vander Wal, 2010) was used to measure the extent to which individuals can successfully challenge and replace maladaptive ideas with more balanced and adaptive thinking. For example, “I consider multiple options before making a decision.” Participants were asked to rate the items on a seven-point Likert-type scale. It has been demonstrated adequate reliability and validity among the Chinese sample (Wang et al., 2016). The Cronbach’s alpha coefficient for CFI was 0.92 in our sample.

#### Intolerance of Uncertainty

The 12-item Intolerance of Uncertainty Scale (IUS-12, Carleton et al., 2007; Zhang et al., 2017) was used to assess one’s reactions to uncertainty, ambiguous situations, and the future (e.g., “Unforeseen events upset me greatly”). It was scored on a five-point Likert-type scale ranging from 1 (not at all characteristic of me) to 5 (entirely characteristic of me), with higher scores indicating lower levels of uncertainty tolerance. Good reliability and validity of IUS-12 in Chinese adults have been confirmed (Zhang et al., 2017). The Cronbach’s alpha coefficient for IUS-12 was 0.86 in our sample.

#### Impulse Buying

The 20-item Impulse Buying Tendency Scale (IBT, Verplanken and Herabadi, 2001; Liu et al., 2019) was used to measure impulsive buying behavior. This scale consists of two dimensions, namely cognitive facet (e.g., “I like to compare different brands before I buy one”) and affective facet (e.g., “I sometimes feel guilty after having bought something”). Each dimension has 10 items. Participants were asked to rate the items on a seven-point Likert-type scale (1 = totally disagree and 7 = totally agree). The validity of the scale has been confirmed among the Chinese population (Liu et al., 2019). The Cronbach’s alpha coefficients for the cognitive subscale was 0.79 and for the affective subscale was 0.83 in our sample.

### Data Analysis

According to a previous study (Neter et al., 1985), the significance of the multicollinearity problems among main study variables was tested in our study. Results showed that the maximum value of Variance Inflation Factor (VIF) was 3.12, indicating no multicollinearity in the current study. Descriptive statistics, correlation analyses, independent sample *t*-test, and one-way ANOVA were conducted by SPSS 24.0 Structural equation modeling (SEM) was performed by AMOS 20.0 to explore the mediating roles of intolerance of uncertainty and cognitive flexibility. All continuous variables were mean centered before entering into the structural equation modeling. Statistical significance was set at *p* < 0.05 in this study.

## Results

### Preliminary Analysis

Mean score of undergraduates’ impulse buying was (68.66 ± 14.38), which was significantly higher than that of the student sample reported by Lucas and Koff (2014) (56.20 ± 14.50, *t* = 23.19, *p* < 0.001, Cohen’s *d* = 0.86). Paired sample *t*-test showed that participants in our study scored significantly higher on the affective facet of impulse buying than on the cognitive facet (36.35 ± 8.88 vs. 32.31 ± 7.84, *t* = 12.61, *p* < 0.001, Cohen’s *d* = 0.48).

### Effects of Gender and Economic Status

Univariate two-way ANOVA tests were conducted to examine the effects of gender and economic status on two components of impulse buying tendency, respectively. Results showed that the main effect of gender is significant on IBT_A [*F*_(1_,_710)_ = 8.56, *p* = 0.004 < 0.01]. Further analyses found that female students exhibited higher scores of affective facet of impulse buying (for female: 36.82 ± 8.63, for male: 34.30 ± 9.64, *p* = 0.004 < 0.001, Cohen’s *d* = 0.27). While the main effect of economic status [*F*_(2_,_710)_ = 1.04, *p* = 0.354 > 0.05] and the interaction effect of gender × economic status on IBT_A [*F*_(2_,_710)_ = 0.08, *p* = 0.922 > 0.05] did not reach statistical significance. For IBT_C, similar results were obtained. The main effect of gender is significant on IBT_C [*F*_(1_,_710)_ = 16.25, *p* < 0.001] and female students exhibited higher scores of cognitive facet of impulse buying than male students (for female: 32.98 ± 7.55, for male: 29.36 ± 8.45, *p* < 0.001, Cohen’s *d* = 0.45). The main effect of economic status [*F*_(2_,_710)_ = 2.04, *p* = 0.130 > 0.05] and the interaction effect of gender × economic status on IBT_C [*F*_(2_,_710)_ = 2.85, *p* = 0.059 > 0.05] were not significant.

### Associations of Study Variables

To explore the associations among the concerned variables, Pearson correlation analyses were performed (see Table 1). Results showed that two dimensions of impulse buying tendency were positively related to anxiety, depression, and intolerance of uncertainty, while all these variables were negatively related to cognitive flexibility (*ps* < 0.001). In addition, both anxiety and depression were positively related to intolerance of uncertainty and negatively related to cognitive flexibility (*ps* < 0.001). Therefore, the initial hypotheses (1) and (2) were totally supported.

**TABLE 1:** Correlations among the study variables (*n* = 716).

	Mean ± SD	Anxiety	Depression	Intolerance of uncertainty	Cognitive flexibility	IBT_C
Anxiety	4.07 ± 3.54	–				
Depression	4.53 ± 4.14	0.81***	–			
Intolerance of uncertainty	36.02 ± 7.09	0.50***	0.45***	–		
Cognitive flexibility	101.38 ± 14.96	−0.37***	−0.38***	−0.38***	–	
IBT_C	32.31 ± 7.84	0.15***	0.16***	0.16***	−0.31***	–
IBT_A	36.35 ± 8.88	0.29***	0.29***	0.53***	−0.22***	0.48***

***p < 0.001. IBT_C, the cognitive facet of impulse buying tendency; IBT_A, the affective facet of impulse buying tendency.

### Structural Equation Modeling (SEM)

In order to test the possible mediating roles of intolerance of uncertainty and cognitive flexibility between negative emotions and impulse buying, the hypothesized model in Figure 1 was estimated. Considering the significant influence of gender on IBT_A and IBT_C, gender was controlled as a demographic variable in the SEM. The results revealed that six regression coefficients were not significant [IBT_C < — anxiety (*b* = −0.04, *p* = 0.514), IBT_A < — anxiety (*b* = −0.05, *p* = 0.408), IBT_C < — depression (*b* = 0.07, *p* = 0.283), IBT_A < — depression (*b* = 0.10, *p* = 0.074), IBT_A < — cognitive flexibility (*b* = 0.01, *p* = 0.791), and IBT_C < — intolerance of uncertainty (*b* = 0.06, *p* = 0.176)]. Therefore, these six pathways were removed and the modified model was recalculated. Results revealed that the modified model fit the data well with χ^2^/*df* = 1.790. The goodness-of-fit indices of this model were as follows: NFI = 0.991, TLI = 0.999, GFI = 0.996, SRMR = 0.031, and RMSEA = 0.033.

**FIGURE 2:**
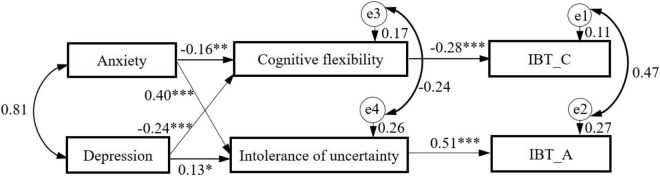
The mediation model between anxiety, depression, and impulse buying with intolerance of uncertainty and cognitive flexibility as mediators, after controlling for gender. Standardized regression coefficients are presented. IBT_C, the cognitive facet of impulse buying tendency; IBT_A, the affective facet of impulse buying tendency. *p < 0.05, ^**^p < 0.01, ^***^p < 0.001.

As shown in Figure 2, IBT_C was directly predicted by cognitive flexibility (β = −0.28, *p* < 0.001), while it was indirectly predicted by anxiety (β = 0.05, *p* = 0.013 < 0.01) and depression (β = 0.07, *p* < 0.001) via cognitive flexibility in this modified model. Meanwhile, IBT_A was directly predicted by intolerance of uncertainty (β = 0.51, *p* < 0.001) and indirectly predicted by anxiety (β = 0.21, *p* = 0.001 < 0.01) and depression (β = 0.06, *p* = 0.022 < 0.05) via intolerance of uncertainty. In addition, cognitive flexibility was negatively influenced by anxiety (β = −0.16, *p* = 0.005 < 0.01) and depression (β = −0.24, *p* < 0.001). By contrast, intolerance of uncertainty was positively influenced by anxiety (β = 0.40, *p* < 0.001) and depression (β = 0.13, *p* = 0.023 < 0.05).

Subsequently, bootstrapping procedures in AMOS were used to test the significance of the mediation model. According to the previous finding (Mackinnon et al., 2004), 2000 bootstrapping samples were generated from the original data set by random sampling. Results showed that anxiety (*b* = 0.05, 95% CI: 0.02–0.08) and depression (*b* = 0.07, 95% CI: 0.04–0.11) had significant indirect effects on cognitive facet of impulse buying via cognitive flexibility, and they also exerted significant indirect effects (*b* = 0.21, 95% CI: 0.14–0.27 for anxiety; *b* = 0.06, 95% CI: 0.01–0.13 for depression) on the affective facet of impulse buying via intolerance of uncertainty. Accordingly, our initial hypothesis (3) that intolerance of uncertainty and cognitive flexibility mediate the relationships between anxiety, depression, and impulse buying was partially confirmed. This modified model accounted for 10.5% of IBT_C variance, 27.2% of IBT_A variance, 16.8% of cognitive flexibility variance, and 25.9% of intolerance of uncertainty variance.

## Discussion

The unprecedented COVID-19 epidemic swept the whole world and brought out an inestimable impact on the global economy and people’s daily life (Balsamo and Carlucci, 2020; Carlucci et al., 2020; El Keshky et al., 2020). Due to the public uncertainty and psychological pressure triggered by the COVID-19 pandemic (Khademian et al., 2021; Zhuo et al., 2021), people’s consumption patterns have also greatly changed. Several studies have shown that consumers reported making more impulse purchases during the COVID-19 pandemic than before (Xiao et al., 2020; Hu et al., 2021; Naeem, 2021). It is very necessary to reshape individual healthy consumption psychology and rational consumption behavior. The main purpose of this study was to explore the underlying mechanism of impulse buying through the lens of the emotion-cognition-behavior loop among Chinese undergraduates.

Impulse buying often goes along a wide range of negative impacts (such as excessive credit and financial distress) and a state of negative affect such as guilt and regret (Li et al., 2015). Therefore, young people’s irrational impulse buying behaviors should be concerned. Our study found that the levels of impulse buying among Chinese undergraduates during the COVID-19 pandemic were higher than those of other undergraduates samples (Lucas and Koff, 2014; Liu et al., 2019). Our results also showed that the scores of undergraduates on the affective facet of impulse buying were significantly higher than those on the cognitive facet. This agrees with previous findings that individuals often experience many affective conflicts before, during, and after shopping (Lucas and Koff, 2014, 2017). To cope with impulse buying during COVID-19, both internal and external aspects should be considered. As proposed by Gupta et al. (2021), government and policymakers should ensure an efficient supply chain management which can help in easing consumers’ anxiety and depression. At the same time, from the perspective of individual mental health, changes of individual intrinsic factors may be the key to solve the problem.

In consistent with previous studies (Silvera et al., 2008; Lucas and Koff, 2014), this study revealed that females exhibited higher levels of two dimensions of impulse buying, compared with male students. Our results further confirmed the fact that females tend to be driven by the affective aspects and are more likely to participate in unplanned buying (Coley and Burgess, 2003; Chang et al., 2014). As hypothesized, we found that more impulse buying behaviors went along with more anxiety and depression symptoms. This finding well agrees with the viewpoint proposed by previous researchers (Badgaiyan et al., 2016) that individuals with emotional instability, anxiety, moodiness, and irritability reported more impulsive tendencies. Meanwhile, impulse buying was associated with intolerance of uncertainty and cognitive inflexibility, which adds credibility to the viewpoint that impulsive buying is often associated with cognitive vulnerability (Youn and Faber, 2000; Badgaiyan et al., 2016).

The mediation model revealed that anxiety and depression did not directly influence affective impulse buying, but had indirect effects on affective impulse buying through intolerance of uncertainty. This perhaps reflects the fact that individuals reporting more negative emotion usually have inability or unwillingness to expend the energy necessary to take action in the face of uncertainty (Jensen et al., 2016). Consequently, they tend to employ ineffective coping behaviors (Iyer et al., 2019) and experience increased affective response (Simmons et al., 2008), i.e., the affective facet of impulsive behaviors. In addition, cognitive flexibility fully mediated the relationships between anxiety, depression, and cognitive impulse buying. This is consistent with the previous studies (Soltani et al., 2013; Lee and Orsillo, 2014) stating that anxiety and depression are characterized by an inflexible style in the cognitive processing of information, which can increase risk behaviors in decision-making. Accordingly, this study may provide new insight into how anxiety and depression link to impulse buying, by demonstrating the fact that intolerance of uncertainty and cognitive flexibility mediate the relationships between anxiety, depression, and impulse buying during the COVID-19 pandemic. In addition, the effects of intolerance of uncertainty and cognitive flexibility on impulse buying were found to be above and beyond the impact of negative emotions. Consistent with the finding by Verplanken and Herabadi (2001), different personality bases of the two components of impulse buying tendency were also found in this study. Specifically, in addition to being affected by both anxiety and depression, our study found for the first time that the affective facet could be predicted by personal tolerance to uncertainty while the cognitive impulse buying was closely related to cognitive inflexibility. This suggests that different intervention programs should be formulated for individuals with affective impulse buying and individuals with cognitive impulse buying.

## Theoretical and Practical Implications

The results provided a basis for increasing understanding of impulse buying more likely during the COVID-19 pandemic and shed light on how negative emotions influence impulse buying and provided strong evidence for the close relationship between cognitive characteristics (i.e., intolerance of uncertainty and cognitive flexibility) and impulse buying. Our study illustrated students exhibited more affective impulse buying than cognitive impulse buying due to their experience of a wider range of affect-inducing events during the COVID-19 pandemic. We detected correlations between anxiety, depression, and impulse buying. The hypothesized emotion-cognition-behavior loop was supported by revealing that negative emotions and cognitive characteristics interacted and exhibited their impacts on impulse buying together. This study also suggested that increasing tolerance to the uncertainty of internal/external environment and learning flexible coping styles may help to reduce young people’s impulse buying behaviors, thereby affording guidance for individuals with maladaptive behaviors. These findings offer useful guidance on professional counseling and mental health education. The practitioners can teach students effective strategies to enhance their cognitive flexibility and tolerance to uncertainty, so as to reduce their panic and irrational consumption during the COVID-19 epidemic.

## Conclusion

Considering the changes in personal buying habits and consumption patterns during the COVID-19 pandemic, the present study explored roles of anxiety and depression in triggering impulse buying. Besides that, how individual cognitive characteristics affect individual pattern of impulse buying were also examined among Chinese undergraduates. We found that undergraduates scored significantly higher on the affective facet of impulse buying than on the cognitive facet. Female students exhibited more impulse buying tendency than males. Intolerance of uncertainty fully mediated the relationships between anxiety, depression, and affective impulse buying. While cognitive flexibility fully mediated the relationships between anxiety, depression, and cognitive impulse buying. These results confirmed the two components of impulse buying tendency have different cognitive characteristics. Future research will be needed to determine the optimal format and content of intervention programs from the perspective of improving individual cognitive characteristics to reduce impulsive consumption.

## Limitations and Future Directions

Some shortcomings of the current study should be emphasized. Firstly, the cross-sectional design limits its ability to infer the causal relationship between study variables. For example, there may be a reversed causal relationship between impulse buying and negative emotions. In other words, people with a lower frequency of impulse buying may be inherently less likely to have anxiety or depression symptoms. Therefore, further longitudinal investigation is necessary. Secondly, the imbalance of the gender ratio hinders the generalization of this result. Our findings need to be further verified in other populations. Future studies could thus carry out more robust designs and even potentially infer causality between negative emotions and impulsive buying.

## Data Availability Statement

The raw data supporting the conclusions of this article will be made available by the authors, without undue reservation.

## Ethics Statement

The studies involving human participants were reviewed and approved by the Ethics Committee of Yangtze Normal University. The patients/participants provided their written informed consent to participate in this study.

## Author Contributions

YY designed the study, collected the data, analyzed the data, and wrote the manuscript independently.

## Conflict of Interest

The author declares that the research was conducted in the absence of any commercial or financial relationships that could be construed as a potential conflict of interest.

## Publisher’s Note

All claims expressed in this article are solely those of the authors and do not necessarily represent those of their affiliated organizations, or those of the publisher, the editors and the reviewers. Any product that may be evaluated in this article, or claim that may be made by its manufacturer, is not guaranteed or endorsed by the publisher.

## References

[B1] Ahmed R. R. Streimikiene D. Rolle J. Duc P. A. (2020). The COVID-19 Pandemic and the antecedants for the impulse buying behavior of US citizens. *J. Competitiveness* 12 5–27. 10.7441/joc.2020.03.01

[B2] Akram U. Hui P. Kaleem Khan M. Tanveer Y. Mehmood K. Ahmad W. (2018a). How website quality affects online impulse buying: moderating effects of sales promotion and credit card use. *Asia Pac. J. Market. Lo.* 30 235–256. 10.1108/apjml-04-2017-0073

[B3] Akram U. Khan M. K. Hui P. Tanveer Y. Akram Z. (2018b). Development of e-commerce: factors influencing online impulse shopping in China. *J. Electron. Commer. Or.* 16 29–47. 10.4018/jeco.2018040102

[B4] Badgaiyan A. J. Verma A. Dixit S. (2016). Impulsive buying tendency: measuring important relationships with a new perspective and an indigenous scale. *IIMB Manag. Rev.* 28 186–199. 10.1016/j.iimb.2016.08.009

[B5] Balsamo M. Carlucci L. (2020). Italians on the age of COVID-19: the self-reported depressive symptoms through web-based survey. *Front. Psychol.* 11:569276. 10.3389/fpsyg.2020.569276 33178074 PMC7596268

[B6] Carleton R. N. Norton M. A. P. J. Asmundson G. J. G. (2007). Fearing the unknown: a short version of the Intolerance of Uncertainty Scale. *J. Anxiety Disord.* 21 105–117. 10.1016/j.janxdis.2006.03.014 16647833

[B7] Carlucci L. D’Ambrosio I. Balsamo M. (2020). Demographic and attitudinal factors of adherence to quarantine guidelines during COVID-19: the Italian model. *Front. Psychol.* 11:559288. 10.3389/fpsyg.2020.559288 33192820 PMC7609562

[B8] Çelik S. Köse G. G. (2021). Mediating effect of intolerance of uncertainty in the relationship between coping styles with stress during pandemic (COVID-19) process and compulsive buying behavior. *Prog. Neuropsychopharmacol. Biol. Psychiatry* 110:110321. 10.1016/j.pnpbp.2021.110321 33819541 PMC8579686

[B9] Chamberlain S. R. Fineberg N. A. Blackwell A. D. Robbins T. W. Sahakian B. J. (2006). Motor inhibition and cognitive flexibility in obsessive-compulsive disorder and trichotillomania. *Am. J. Psychiatry* 163 1282–1284. 10.1176/appi.ajp.163.7.1282 16816237

[B10] Chang H. J. Yan R. N. Eckman M. (2014). Moderating effects of situational characteristics on impulse buying. *Int. J. Retail Distrib.* 42 289–314. 10.1108/IJRDM-04-2013-0074

[B11] Clark D. M. Fairburn C. G. (1997). *Science and Practice of Cognitive Behaviour Therapy.* Oxford: Oxford University Press.

[B12] Clemens V. Deschamps P. Fegert J. M. Anagnostopoulos D. Bailey S. Doyle M. (2020). Potential effects of “social” distancing measures and school lockdown on child and adolescent mental health. *Eur. Child Adolesc. Psychiatry* 29 739–742. 10.1007/s00787-020-01549-w 32447569 PMC7245163

[B13] Coley A. Burgess B. (2003). Gender differences in cognitive and affective impulse buying. *J. Fash. Mark. Manag.* 7 282–295. 10.1108/13612020310484834

[B14] Denfeld Q. Erickson E. Valent A. Villasana L. Zhang Z. Myatt L. (2020). COVID-19: Challenges and lessons learned from early career investigators. *J. Womens Health (Larchmt)* 29 752–754. 10.1089/jwh.2020.8552 32469620 PMC7307672

[B15] Dennis J. P. Vander Wal J. S. (2010). The Cognitive Flexibility Inventory: instrument development and estimates of reliability and validity. *Cognitive Ther. Res.* 34 241–253. 10.1007/s10608-009-9276-4

[B16] Deveney C. M. Deldin P. J. (2006). A preliminary investigation of cognitive flexibility for emotional information in major depressive disorder and non-psychiatric controls. *Emotion* 6 429–437. 10.1037/1528-3542.6.3.429 16938084

[B17] Dugas M. J. Schwartz A. Francis K. (2004). Intolerance of uncertainty, worry, and depression. *Cognitive Ther. Res.* 28 835–842. 10.1007/s10608-004-0669-0

[B18] El Keshky M. E. S. Basyouni S. S. Al Sabban A. M. (2020). Getting through COVID-19: the pandemic’s impact on the psychology of sustainability, quality of life, and the global economy - a systematic review. *Front. Psychol.* 11:585897. 10.3389/fpsyg.2020.585897 33281683 PMC7688781

[B19] Fredrickson B. L. (2001). The role of positive emotions in positive psychology: the broaden-and-build theory of positive emotions. *Am. Psychol*. 56 218–226. 10.1037/0003-066x.56.3.218 11315248 PMC3122271

[B20] Guastella A. J. Dadds M. R. (2009). Sequential growth in cognitive-behavioral emotion-processing: a laboratory study. *Cogn. Ther. Res.* 33 368–374. 10.1007/s10608-008-9199-5

[B21] Gupta R. Nair K. Radhakrishnan L. (2021). Impact of COVID-19 crisis on stocking and impulse buying behaviour of consumers. *Int. J. Soc. Econ.* 48 1794–1809. 10.1108/IJSE-03-2021-0163

[B22] Haq M. A. Abbasi S. (2016). Indirect impact of hedonic consumption and emotions on impulse purchase behavior: a double mediation model. *J. Manage. Sci.* 3 108–122. 10.20547/jms.2014.1603202

[B23] Hollingsworth D. W. Gauthier J. M. McGuire A. P. Peck K. R. Hahn K. S. Connolly K. M. (2018). Intolerance of uncertainty mediates symptoms of PTSD and depression in African American veterans with comorbid PTSD and substance use disorders. *J. Black Psychol.* 44 667–688. 10.1177/0095798418809201

[B24] Hu Y. Q. Xie P. Wang Y. Huang Z. X. Wu Y. T. Sun H. L. (2021). The influence of COVID-19 on irrational consumption behavior in a Chinese sample: based on a serial mediating model. *Front. Psychol.* 12:718797. 10.3389/fpsyg.2021.718797 34764908 PMC8576605

[B25] Islam T. Pitafi A. H. Arya V. Wang Y. Akhater N. Mubarik S. (2021). Panic buying in the COVID-19 pandemic: a multi-country examination. *J. Retail. Consum. Serv.* 59:102357. 10.1016/j.jretconser.2020.102357

[B26] Iyer G. R. Blut M. Xiao S. H. Grewal D. (2019). Impulse buying: A meta-analytic review. *J. Acad. Market. Sci.* 48 384–404. 10.1007/s11747-019-00670-w

[B27] Jensen D. Cohen J. N. Mennin D. S. Fresco D. M. Heimberg R. G. (2016). Clarifying the unique associations among intolerance of uncertainty, anxiety, and depression. *Cogn. Behav. Ther.* 45 431–444. 10.1080/16506073.2016.1197308 27314213 PMC5045801

[B28] Johnco C. Wuthrich V. M. Rapee R. M. (2014). Reliability and validity of two self-report measures of cognitive flexibility. *Psychol. Assess.* 26 1381–1387. 10.1037/a0038009 25265414

[B29] Kalia V. Knauft K. Hayatbini N. (2020). Cognitive flexibility and perceived threat from COVID-19 mediate the relationship between childhood maltreatment and state anxiety. *PLoS One* 15:e0243881. 10.1371/journal.pone.0243881 33306748 PMC7732062

[B30] Khademian F. Delavari S. Koohjani Z. Khademian Z. (2021). An investigation of depression, anxiety, and stress and its relating factors during COVID-19 pandemic in Iran. *BMC Public Health* 21:275. 10.1186/s12889-021-10329-3 33535992 PMC7856614

[B31] Kroenke K. Spitzer R. L. Williams J. B. (2001). The PHQ-9: validity of a brief depression severity measure. *J. Gen. Intern. Med.* 16 606–613. 10.1046/j.1525-1497.2001.016009606.x 11556941 PMC1495268

[B32] Lee J. K. Orsillo S. M. (2014). Investigating cognitive flexibility as a potential mechanism of mindfulness in generalized anxiety disorder. *J. Behav. Ther. Exp. Psy.* 45 208–216. 10.1016/j.jbtep.2013.10.008 24239587

[B33] Leverin A. Liljander V. (2006). Does relationship marketing improve customer relationship satisfaction and loyalty? *Int. J. Bank Mark.* 24 232–251. 10.1108/02652320610671333

[B34] Li Z. F. Deng S. Moutinho L. (2015). The impact of experience activities on tourist impulse buying: an empirical study in China. *Asia Pac. J. Tour. Res.* 20 191–209. 10.1080/10941665.2013.877043

[B35] Liang L. Gao T. Ren H. Cao R. Qin Z. Hu Y. (2020). Post-traumatic stress disorder and psychological distress in Chinese youths following the COVID-19 emergency. *J. Health Psychol.* 25 1164–1175. 10.1177/1359105320937057 32627606 PMC7342938

[B36] Lin C. T. Chen C. W. Wang S. J. Lin C. C. (2018). The influence of impulse buying toward consumer loyalty in online shopping: a regulatory focus theory perspective. *J. Amb. Intel. Hum. Comp.* 1–11. 10.1007/s12652-018-0935-8

[B37] Liu P. He J. Li A. (2019). Upward social comparison on social network sites and impulse buying: a moderated mediation model of negative affect and rumination. *Comput. Hum. Behav.* 96 133–140. 10.1016/j.chb.2019.02.003

[B38] Lucas M. Koff E. (2014). The role of impulsivity and of self-perceived attractiveness in impulse buying in women. *Pers. Indiv. Differ.* 56 111–115. 10.1016/j.paid.2013.08.032

[B39] Lucas M. Koff E. (2017). Body image, impulse buying, and the mediating role of negative affect. *Pers. Indiv. Differ.* 105 330–334. 10.1016/j.paid.2016.10.004

[B40] Mackinnon D. P. Lockwood C. M. Williams J. (2004). Confidence limits for the indirect effect: distribution of the product and resampling methods. *Multivar. Behav. Res.* 39 99–128. 10.1207/s15327906mbr3901_4 PMC2821115 20157642

[B41] Morriss J. Christakou A. van Reekum C. M. (2016). Nothing is safe: intolerance of uncertainty is associated with compromised fear extinction learning. *Biol. Psychol.* 121 187–193. 10.1016/j.biopsycho.2016.05.001 27178640 PMC5154327

[B42] Naeem M. (2021). Understanding the customer psychology of impulse buying during COVID-19 pandemic: implications for retailers. *Int. J. Retail Distrib.* 49 377–393. 10.1108/IJRDM-08-2020-0317

[B43] Neter J. Wassermanm W. Kutner M. H. (1985). *Applied Linear Statistical Models: Regression, Analysis of Variance, and Experimental Designs*, 2nd Edn. Homewood, IL: Richard D. Irwin, Inc.

[B44] Qu S. Sheng L. (2015). Diagnostic test of screening generalized anxiety disorders in general hospital psychological department with GAD-7. *Chin. Ment. Health J.* 29 939–944. 10.3969/j.issn.1000-6729.2015.12.010

[B45] Rende B. (2000). Cognitive flexibility: theory, assessment, and treatment. *Semin. Speech Lang*. 21 121–132. 10.1055/s-2000-7560 10879545

[B46] Satici B. Saricali M. Satici S. A. Griffiths M. D. (2020). Intolerance of uncertainty and mental wellbeing: serial mediation by rumination and fear of COVID-19. *Int. J. Ment Health Addiction.* [Epub ahead of print]. 10.1007/s11469-020-00305-0 32427165 PMC7228430

[B47] Sheth J. (2020). Impact of COVID-19 on consumer behaviour: will the old habits return or die? *J. Bus. Res.* 117 280–283. 10.1016/j.jbusres.2020.05.059 32536735 PMC7269931

[B48] Silvera D. H. Lavack A. M. Kropp F. (2008). Impulse buying: The role of affect, social influence, and subjective wellbeing. *J. Consum. Mark.* 25 23–33. 10.1108/07363760810845381

[B49] Simmons A. Matthews S. C. Paulus M. P. Stein M. B. (2008). Intolerance of uncertainty correlates with insula activation during affective ambiguity. *Neurosci. Lett.* 430 92–97. 10.1016/j.neulet.2007.10.030 18079060 PMC2692473

[B50] Soltani E. Shareh H. Bahrainian S. A. Farmani A. (2013). The mediating role of cognitive flexibility in correlation of coping styles and resilience with depression. *Pajoohandeh J.* 18 88–96.

[B51] Spitzer R. L. Kroenke K. Williams J. B. W. Löwe B. (2006). A brief measure for assessing generalized anxiety disorder: The GAD-7. *Arch. Intern. Med.* 166 1092–1097. 10.1001/archinte.166.10.1092 16717171

[B52] Verplanken B. Herabadi A. (2001). Individual differences in impulse buying tendency: Feeling and no thinking. *Eur. J. Personal.* 15 S71–S83. 10.1002/per.423

[B53] Wang S. Liu Y. Du Y. Wang X. (2021). Effect of the COVID-19 pandemic on consumers’ impulse buying: the moderating role of moderate thinking. *Int. J. Environ. Res. Public Health* 18:11116. 10.3390/ijerph182111116 34769636 PMC8583521

[B54] Wang Y. Yang Y. Xiao W. T. Su Q. (2016). Validity and reliability of the Chinese version of the Cognitive Flexibility Inventory in college students. *Chin. Ment. Health J.* 30 58–63. 10.3969/j.issn.1000-6729.2016.01.012

[B55] Xiao H. Zhang Z. Zhang L. (2020). A diary study of impulsive buying during the COVID-19 pandemic. *Curr. psychol*. [Epub ahead of print]. 10.1007/s12144-020-01220-2 33250615 PMC7682774

[B56] Youn S. Faber R. J. (2000). Impulse buying: its relation to personality traits and cues. *Adv. Consum. Res.* 27 179–185.

[B57] Yu Y. J. Yu Y. J. Hu J. X. (2021). COVID-19 among Chinese high school graduates: Psychological distress, growth, meaning in life and resilience. *J. Health Psychol.* 27:1057–1069. 10.1177/1359105321990819 33541149 PMC8685742

[B58] Yu Y. J. Yu Y. J. Lin L. G. (2020). Anxiety and depression aggravate impulsiveness: The mediating and moderating role of cognitive flexibility. *Psychol. Health Med.* 25 25–36. 10.1080/13548506.2019.1601748 30947527

[B59] Zhang Y. J. Song J. B. Gao Y. T. Wu S. J. Song L. Miao D. M. (2017). Reliability and validity of the Intolerance of Uncertainty Scale-Short form in university students. *Chin. J. Clin. Psychol.* 25 285–288. 10.16128/j.cnki.1005-3611.2017.02.020

[B60] Zhang Y. L. Liang W. Chen Z. M. Zhang H. M. Zhang J. H. (2013). Validity and reliability of Patient Health Questionnaire-9 and Patient Health Questionnaire-2 to screen for depression among college students in China. *Asia-Pac. Psychiat.* 5 268–275. 10.1111/appy.12103 24123859

[B61] Zhuo L. Wu Q. Le H. Li H. Tao H. (2021). COVID-19-related intolerance of uncertainty and mental health among back-to-school students in Wuhan: The moderation effect of social support. *Int. J. Env. Res. Pub. He.* 18:981. 10.3390/ijerph18030981 33499409 PMC7908243

